# Pharmacist-Led Medication Evaluation Considering Pharmacogenomics and Drug-Induced Phenoconversion in the Treatment of Multiple Comorbidities: A Case Report

**DOI:** 10.3390/medicina57090955

**Published:** 2021-09-10

**Authors:** Nicole Marie Del Toro-Pagán, Adriana Matos, David Thacker, Jacques Turgeon, Nishita Shah Amin, Veronique Michaud

**Affiliations:** 1 Office of Translational Research and Residency Programs, Tabula Rasa HealthCare, Moorestown, NJ 08057, USA; npagan@trhc.com (N.M.D.T.-P.); amatos@carekinesis.com (A.M.); namin@carekinesis.com (N.S.A.); 2 Precision Pharmacotherapy Research & Development Institute, Tabula Rasa HealthCare, Orlando, FL 32827, USA; dthacker@trhc.com (D.T.); jturgeon@trhc.com (J.T.); 3 Faculty of Pharmacy, Université de Montréal, Montréal, QC H3C 3J7, Canada

**Keywords:** antidepressants, β-blockers, case report, clinical decision support system (CDSS), CYP2D6, depression, opioids, pain, pharmacogenetics, pharmacogenomics (PGx)

## Abstract

Pharmacogenomic (PGx) information can guide drug and dose selection, optimize therapy outcomes, and/or decrease the risk of adverse drug events (ADEs). This report demonstrates the impact of a pharmacist-led medication evaluation, with PGx assisted by a clinical decision support system (CDSS), of a patient with multiple comorbidities. Following several sub-optimal pharmacotherapy attempts, PGx testing was recommended. The results were integrated into the CDSS, which supported the identification of clinically significant drug–drug, drug–gene, and drug–drug–gene interactions that led to the phenoconversion of cytochrome P450. The pharmacist evaluated PGx results, concomitant medications, and patient-specific factors to address medication-related problems. The results identified the patient as a CYP2D6 intermediate metabolizer (IM). Duloxetine-mediated competitive inhibition of CYP2D6 resulted in phenoconversion, whereby the patient’s CYP2D6 phenotype was converted from IM to poor metabolizer for CYP2D6 co-medication. The medication risk score suggested a high risk of ADEs. Recommendations that accounted for PGx and drug-induced phenoconversion were accepted. After 1.5 months, therapy changes led to improved pain control, depression status, and quality of life, as well as increased heart rate, evidenced by patient-reported improved sleep patterns, movement, and cognition. This case highlights the pharmacist’s role in using PGx testing and a CDSS to identify and mitigate medication-related problems to optimize medication regimen and medication safety.


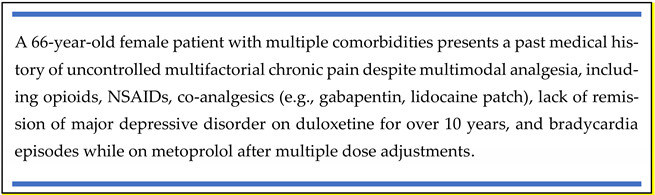


## 1. Introduction

Environmental, physiological, and psychological factors, as well as comorbidities and genetic variability, have been shown to affect interpatient variability in drug disposition and response [1]. Pharmacogenomics (PGx) is the study of human genome variants that impact drug response via variations in pharmacokinetic or pharmacodynamic parameters [2]. Therefore, PGx testing can support the identification of drug–gene interactions (DGIs) and drug–drug–gene interactions (DDGIs). DGIs involve a drug and a variation in a gene that codes for a protein, such as cytochrome P450 (CYP) isoenzymes (e.g., citalopram and CYP2C19), a receptor (e.g., metoprolol and adrenoceptor beta 1 (ADRB1)) or a transporter (simvastatin and solute carrier organic anion transporter 1B1 (SLCO1B1, previously referred to OATP1B1)) [3]. The superimposition of a drug–drug interaction (DDI) on a DGI can result in a DDGI, which frequently induces phenoconversion [3]. Phenoconversion is the ability of intrinsic (e.g., inflammation) [4,5] or extrinsic factors, such as drugs, to modify a genotype-predicted phenotypic expression [6]. For instance, a mismatch between the predicted phenotype from the determined *CYP2C19* genotype and the observed CYP2C19 activity has been reported in patient with type 2 diabetes due to low levels of pro-inflammatory cytokines [7]. Similarly, a drug may induce CYP phenoconversion, and an individual identified as a CYP2D6 normal metabolizer (NM) with a *1|*1 genotype will be phenoconverted into a poor metabolizer (PM) while taking quinidine, a potent CYP2D6 inhibitor. Considering the two previously mentioned conditions, the metabolism of CYP2C19 or CYP2D6 substrates would be altered under such conditions, which may result in an increased risk of inappropriate response to substrates of these enzymes. To mitigate the effect of these DGIs and DDGIs, organizations such as the Clinical Pharmacogenetics Implementation Consortium (CPIC) and the Dutch Pharmacogenetics Working Group (DPWG) have developed guidance on drug and dose selection for certain drug–gene pairs (e.g., duloxetine and CYP2D6, hydrocodone and CYP2D6, metoprolol and CYP2D6) based on current clinical evidence [8].

Large interindividual differences exist in response to analgesic therapy agents, such as prodrug opioids activated by CYP2D6 (e.g., codeine, tramadol, hydrocodone, oxycodone) [9]. The presence of variants in the *CYP2D6* gene can contribute to variability in opioid response in terms of efficacy and/or risk of adverse drug events (ADEs). The opioid receptor µ1 (*OPRM1*) and catechol-O-methyltransferase (*COMT*) gene variants have also been studied for their potential to affect opioid pharmacodynamic response [10]. While CPIC provides CYP2D6 genotype/phenotype-based recommendations for codeine, tramadol, and hydrocodone, no recommendations are currently available for dosing opioids based on either the *OPRM1* or *COMT* genotype due to the lack of consistent evidence [11]. The prevalence of CYP450 DDIs among patients with chronic low back pain on long-term opioid therapy has been estimated to be 27%, which can further impact the response variability observed due to a higher risk of DDGIs [12]. The vast majority of the reported DDIs in an older adult population cohort were associated with medications known to inhibit the CYP2D6 enzyme [12].

Integrating PGx into clinical practice could support antidepressant drug and dose selection. Currently, the antidepressant medication selection for each patient is dependent upon trial-and-error treatment strategies considering endophenotype, clinician preferences and clinical experience, and the patient’s past medication history [13]. Implementation of a PGx decision support tool for antidepressant treatment may enhance the prediction of treatment response and the capability to foresee potential adverse side effects, minimizing prolonged suffered and adverse sequelae. PGx can support narrowing down the antidepressant drug and dose options, potentially leading to decreased risk of antidepressant-related toxicity and/or to achieve remission faster [14,15]. Variations in the *CYP2C19* and/or *CYP2D6* genes contributes to the variability observed in antidepressant response [16]. CPIC and DPWG provide genotype/phenotype-based recommendations for multiple selective serotonin reuptake inhibitors (SSRIs), serotonin-norepinephrine reuptake inhibitors (SNRIs), and tricyclic antidepressants [17,18,19]. The glutamate ionotropic receptor kainate type subunit 4 (*GRIK4*) and 5-hydroxytryptamine receptor 2A (*HTR2A*) gene variants have been studied for their potential to affect antidepressant pharmacodynamic response. However, no dosing recommendations are currently provided based on either genotype due to the dearth of consistent evidence [20].

Polymorphisms in CYP2D6 are also associated with altered β-blocker metabolism [21]. CYP2D6 IMs and PMs typically experience greater reduction in heart rate, and some patients develop symptomatic bradycardia [22]. The *ADRB1* and G protein-coupled receptor kinase 5 (*GRK5*) gene variants have been studied for their potential to affect β-blockers’ pharmacodynamic response. However, no dosing recommendations are currently provided based on either genotype due to the lack of consistent evidence [21].

Unquestionably, applying PGx can be beneficial in certain cases. For example, studies of patients suffering from chronic pain and depression have revealed that pain- and depression-induced neuroplasticity changes and neurobiological mechanism changes can be closely interrelated [23]. Lack of control of these conditions can result in an enormous burden to the patient, caregivers, and healthcare system, resulting in increased costs [24,25]. Uncontrolled pain also reduces functional capacity and quality of life for patients, such as those living with obesity [26]. Patients diagnosed with chronic pain living with obesity are more likely to present with depressive symptoms than patients who are normal- or overweight [27]. It should be noted that depression in the older adult population has been associated with longer length of illness, more frequent major depressive disorder (MDD) recurrences, and a greater risk of comorbidities [28]. In this population, depression has been identified as one of the psychiatric illness most closely associated with suicide [29]. In 2012, a literature review reported that patients who have an inadequate response to their antidepressant regimen are expected to spend approximately USD 10,000 more annually on healthcare-related expenses than patients who have an adequate response [30]. Uncontrolled pain in the older adult population results in an annual cost of approximately USD 61.2 billion [31], while loss of productivity for patients and caregivers due to lack of pain control has been calculated to be USD 300 billion [32]. There is potential for cost savings in these populations.

The Program of All-inclusive Care for the Elderly (PACE) receives capitated reimbursements on behalf of Medicare and Medicaid (a joint, federal and state program in the U.S.) for participants 55 years or older. PACE participants have a team of health care professionals collaborating to ensure that nursing home level coordinated care is provided in the home setting. The PACE model enables collaboration between pharmacists and other healthcare practitioners to identify and mitigate medication-related problems. PGx testing is one part of the initiatives implemented to further improve the care of PACE participants [33]. Clinical decision support systems (CDSS) are critical tools for the implementation of PGx into routine patient care and the adoption of PGx recommendations [34]. The proprietary CDSS, MedWise^®^, which has been described previously, incorporates PGx results in combination with the medication regimen to support clinicians with identifying clinically significant DDIs, DGIs, and DDGIs [35]. This CDSS generates a medication risk score (MRS) based on 5 factors, including CYP DDI risk. An increased MRS has been associated with a higher incidence of ADEs, healthcare-related expenditures, emergency department visits, hospitalizations and death [36]. Thus, the objective of this case report is to demonstrate the impact of a pharmacist-led medication evaluation, which incorporated PGx assisted by a CDSS, of a PACE participant with obesity and multiple comorbidities.

## 2. Description of the Case Report

A 66-year-old non-smoker female presented with a past medical history of obesity class III (body mass index = 64 kg/m^2^), uncontrolled chronic pain of multifactorial nature, uncontrolled MDD, hypertension, heart failure, atrial fibrillation, gout, hypothyroidism, type 2 diabetes mellitus, gastroesophageal reflux disease (GERD), insomnia, diarrhea, nausea, and candidiasis. Her medication regimen to treat her multiple comorbidities as prescribed by her primary care physician is described in Table 1. The CDSS generates a medication risk score (MRS) based on the current patient’s drug regimen. The MRS is associated with healthcare outcomes and is the indicator used to measure the risk of adverse drug events associated with a given drug regimen. The MRS for this patient’s drug regimen was 32 with a high-risk sub-score for CYP450 competitive drug interactions and very high-risk sub-score for sedative burden.

The patient’s chief complaints when she enrolled in the PACE program in 2020 were inappropriate control of her depression and poor multifactorial pain control despite numerous trials of antidepressant and analgesic therapies (Table 2). Duloxetine was initially chosen to treat her depression status since it could also provide neuropathic pain relief. However, after being prescribed duloxetine for over 10 years, the patient experienced only minor improvement of MDD symptoms, and despite the use of a multimodal analgesic regimen, the patient continued experiencing uncontrolled pain. The addition of opioids (i.e., tramadol, hydrocodone) to her analgesic regimen was unsuccessful. Of note, while under treatment with metoprolol, the patient was borderline bradycardic per clinical assessment from her primary care physician. Upon review of the patient’s drug regimen, utilizing the proprietary evidenced-based CDSS, the clinical pharmacist recommended conducting a PGx test to guide the optimization of her antidepressant and analgesic regimen. This recommendation was accepted by the primary care physician.

## 3. Results

A DNA sample was collected via buccal swab and analyzed by a Clinical Laboratory Improvement Amendments-certified laboratory (OneOme, Minneapolis, MN, USA). The clinical pharmacist was consulted to interpret the PGx results and complete a multidrug interaction screening. Genotypic results identified the patient as a CYP2D6 IM, with a *1|*4 genotype (Table 3). PGx results were integrated into the CDSS [35]. The clinical pharmacist evaluated the PGx results, concomitant medications, the CDSS-generated MRS, and other patient-specific factors in order to formulate recommendations to the primary care physician for mitigating these medication-related problems to the primary care physician. Although the clinical pharmacist assessed the complete drug regimen, only recommendations relevant to pain, depression, and heart rate control management will be discussed in the context of this case report.

Considering the patient’s CYP2D6 IM status, DGIs were identified affecting the metabolism of hydrocodone, duloxetine, and metoprolol. A CYP2D6 IM has reduced enzyme activity, which alters the clearance of duloxetine and metoprolol and can increase the risk of toxicity associated with these medications. CYP2D6-reduced enzyme activity also decreases the conversion of hydrocodone to hydromorphone and may increase the risk of pharmacotherapy failure and possibly ADEs. Therefore, the clinical pharmacist recommended changing duloxetine to desvenlafaxine, hydrocodone to morphine, and metoprolol to carvedilol. The three recommendations were accepted by the primary care physician.

## 4. Discussion

The CDSS displays duloxetine, metoprolol, and hydrocodone (prodrug) as CYP2D6-substrates (Table 4). As a CYP2D6 IM, we would expect decreased activation of the prodrug, hydrocodone, and increased plasma concentrations of duloxetine and metoprolol due to decreased clearance of these metabolic pathways. When co-administered, duloxetine is expected to competitively inhibit the metabolism of hydrocodone and metoprolol, resulting in drug-induced phenoconversion to PM for these two drugs.

The presence of drug-induced phenoconversion was established based on the CDSS used in this study. This CDSS, supported by clinical and scientific literature, has embedded algorithms that consider several pharmacokinetic properties related to CYP450 metabolic pathways and their respective affinity (patent: WO 2019/089725). The phenotype based on the genotype (derived from PGx results) is combined with CYP450 drug-induced phenoconversion to estimate the patient phenotype for a given drug regimen. Considering that phenoconversion to a PM status was occurring at CYP2D6 for hydrocodone and metoprolol, the clinical pharmacist referred to CPIC guidance for dosing hydrocodone (Table S1) and DPWG guidelines (Table S2) recommendations for dosing metoprolol. The clinical pharmacist chose to recommend morphine, which is not dependent on CYP2D6 metabolism, avoiding DGI and DDGI on this isoenzyme. Additionally, morphine is a hydrophilic opioid; thus, the volume of distribution should not be significantly influenced by excess adipose tissue [39]. Metoprolol exhibits large inter-individual variability in pharmacodynamic response, likely due to its extensive dependence on CYP2D6 (≈75%) for its clearance [40]. Greater area under the curve concentrations and decreased apparent oral clearance in CYP2D6 PMs and IMs translate into differences in heart rate [21]. Additionally, it is worth mentioning that highly lipophilic beta-blockers, such as metoprolol, have been associated with worsening depression symptoms [41]. Highly lipophilic drugs penetrate the blood–brain barrier more readily, which can result in more profound central nervous system effects [42]. This patient did not achieve heart rate control despite multiple attempts to adjust the metoprolol tartrate dose, and she had a diagnosis of heart failure. Therefore, bisoprolol and carvedilol, which are less lipophilic than metoprolol, were the two beta-blocker alternatives considered [43]. The metabolism of bisoprolol is not dependent on CYP2D6; however, it is metabolized by the CYP3A4 enzyme, and it is subject to competitive inhibition at this enzyme by stronger substrates, such as, amlodipine, dronedarone, and loperamide. This could potentially lead to an increased risk of ADEs. On the other hand, the clearance of carvedilol is significantly dependent on CYP2D6 [44]. Current evidence has shown that CYP2D6 PMs and IMs can have up to 2.5 times higher plasma carvedilol plasma concentrations, when comparing to NMs [45]. Despite increased concentrations of carvedilol, researchers have been unable to establish a relationship between the CYP2D6 phenotype and changes in heart rate, blood pressure, or rate of ADEs in patients with heart failure [46,47]. Thus, currently the 2018 update of the DPWG guidelines claim that the effect of the CYP2D6 DGI for carvedilol is likely clinically insignificant [48]. One plausible explanation for this is that, besides its beta-blocker effects, carvedilol also has alpha-blocking properties different from metoprolol [49]. Carvedilol is administered as a racemic mixture and both isomers have been shown to have equally potent alpha blocker properties; however, the S(-)-isomer is the major contributor to the beta-blocker effect [45]. Studies have shown that decreased CYP2D6 metabolism has a more significant impact on the clearance of the R(-)-isomer (alpha blockade) [45]. In this case, the primary care provider opted to prescribe a trial of carvedilol, though it was advised to start with a lower dose and titrate as tolerated.

Upon medication review, it was noted that 10 years of treatment with the SNRI duloxetine did not produce satisfactory depression remission. Achieving depression remission among older adults is a common challenge, as more than 50% of these patients do not appropriately respond to first-line therapies of SSRIs or SNRIs [50]. For duloxetine, DPWG guidelines (Table S3) do not provide CYP2D6 phenotype-based recommendations, as antidepressant plasma concentrations seem to poorly correlate with antidepressant effectiveness. However, phenotype-based recommendations appear to provide more reliable information on the risk of ADEs [51]. Selecting an alternative antidepressant for an elderly patient is challenging due to the higher prevalence of polypharmacy, higher risk of DDIs, reduced medication adherence, and greater risk of ADEs in this population [52]. Because the patient was identified as a CYP2D6 IM, the clinical pharmacist recommended changing duloxetine to desvenlafaxine, which does not depend on CYP2D6 metabolism. As for duloxetine, desvenlafaxine can also contribute to neuropathic pain relief [53].

A follow-up by the pharmacist occurred one and a half months post-implementation of the aforementioned recommendations to assess the analgesic, antidepressant, and heart rate control responses. Changes in therapy led to significantly improved pain, depression, and heart rate control, improving the quality of life of the patient as reported by the primary care physician. This was evidenced by patient reports of improved sleep patterns, movement, and cognition. As a first step in a more holistic medication action plan, the patient’s MRS decreased from 32 to 29. Bankes et al. reported that each point increase in the MRS was associated with an 8.6% higher risk of having one or more ADEs per year, over USD 1000 in additional medical spending a year, 3.2 additional emergency department visits per 100 patients annually, and 2.1 more hospitalizations per 100 patients per year [36]. Ratigan et al. recently reported that although further evidence is needed to demonstrate that lowering the MRS results in a decrease in ADEs or death, interventions among patients on multiple medications that have higher MRS can enhance medication safety [54]. In this case, the decrease in the MRS was attributed to the fact that DDI-associated risk was diminished with the implementation of the clinical interventions.

Limitations must be considered while integrating PGx into routine clinical practice [55]. A lack of universal training to support health care professionals with the interpretation of PGx results and issues related to their incorporation in the electronic health record exists. Other factors, such as ethnicity, sex, smoking status, and comorbidities, can contribute to interpatient variability in medication response in addition to genetic information. This is an iterative approach, and assessing PGx results is only one of the components to complete a comprehensive medication review, because not all medications have PGx recommendations or supporting evidence available. Furthermore, even when limited evidence exists on the impact of genetic variants for some drugs, this does not necessarily indicate that there is a lack of clinical impact for specific patients based on their comprehensive environment and condition. PGx is a relatively new science that is currently experiencing significant growth and has generated much interest among the research community.

## 5. Conclusions

This patient might have achieved pain and depression remission sooner if a comprehensive preemptive PGx testing review and multidrug interaction screening had been performed. PGx testing can help predict tolerability and response [56], thus potentially enabling a more safe, effective, and cost-effective treatment [57]. This case highlights the value of PGx testing supported by CDSS, and the role of pharmacists in identifying medication-related problems and optimizing drug therapy.

## Figures and Tables

**Table 1:** Current patient’s medication list at the time of the PGx testing.

Condition	Medication	Dose	Frequency	Route of Administration
Chronic pain	Hydrocodone/acetaminophen	7.5/325 mg	Four times daily as needed for pain	PO
	Acetaminophen	650 mg	Three times daily as needed for pain	PO
	Gabapentin	600 mg	Three times daily	PO
	Diclofenac 1%	2 g	Four times daily as needed for pain	topical
	Methyl salicylate/menthol/camphor 4%/30%/10%	1 application	Twice daily as needed for muscle pain	topical
	Lidocaine 4%	1 patch	Daily as needed for pain	topical
MDD	Duloxetine	30 mg	Twice daily	PO
Hypertension with heart failure	Amlodipine	5 mg	Daily	PO
	Metoprolol tartrate	100 mg	Twice daily	PO
	Valsartan	320 mg	Daily	PO
	Clonidine	0.1 mg	Daily	PO
	Furosemide	40 mg	Daily	PO
Atrial Fibrillation	Apixaban	5 mg	Twice daily	PO
	Dronedarone	400 mg	Twice daily	PO
Gout	Allopurinol	300 mg	Daily	PO
Hypothyroidism	Levothyroxine	88 mcg	Daily	PO
Type 2 diabetes mellitus	Insulin aspart	26 units	Three times daily before meals	SC
	Insulin glargine	20 units	Daily	SC
GERD	Pantoprazole	40 mg	Daily	PO
Insomnia	Melatonin	5 mg	Daily	PO
Diarrhea	Loperamide	2 mg	Four times daily as needed for diarrhea	PO
Nausea	Ondansetron	4 mg	Four times daily as needed for nausea	PO
Candidiasis	Nystatin	10,000 unit/gram	Four times daily as needed for skin irritation	PO
Supplements	Calcitriol	0.25 mcg	Daily	PO
	Cholecalciferol	2000 units	Daily	PO
	Iron carbonyl/ ascorbic acid	65/125 mg	Daily	PO

Abbreviations: GERD: Gastroesophageal Reflux Disorder; MDD: Major Depression Disorder; PO: Oral; SC, subcutaneous.

**Table 2:** Primary care provider care—reported analgesic and antidepressant trials after PACE enrollment.

Medication	Prior to Nov/2020	Nov/2020	Dec/2020	Jan/2021	Feb/2021 (Post-PGx)
**Analgesic**					
Gabapentin	1200 mg twice daily + 600 mg daily				
				600 mg three times daily	
					300 mg twice daily
Tramadol	50 to 100 mg three times daily as needed				
Hydrocodone/ acetaminophen			5/325 mg four times daily as needed		
			7.5/325 mg four times daily as needed	
Morphine					7.5 to 15 mg three times daily as needed
Acetaminophen		650 mg three times daily as needed			
Lidocaine patch 4% transdermal		1 patch daily as needed			
Diclofenac gel 1% (topical)	2 g four times daily as needed				
Methyl salicylate/menthol/camphor cream 4–30%–10% (topical)	Apply twice daily as needed for muscle pain				
**Antidepressant**					
Duloxetine	60 mg daily + 30 mg at bedtime				
				30 mg twice daily	
Desvenlafaxine					50 mg daily

Shading in Table 2 corresponds to the time frame that medications were prescribed in the patient’s drug regimen.

**Table 3:** Patient’s PGx results.

Gene.	Result	Phenotype
*CYP1A2*	*1F|*1F	Normal Metabolizer (Possible Rapid Metabolizer) ^†^
*CYP2C9*	*1|*1	Normal Metabolizer (NM)
*CYP2C19*	*1|*17	Rapid Metabolizer (RM)
*CYP2D6*	*1|*4	Intermediate Metabolizer (IM)
^‡^ *CYP3A4*	*1|*22	Undetermined
*CYP3A5*	*3|*3	Poor Expresser

Abbreviations: PGx: Pharmacogenomics, CYP: Cytochrome P450. ^†^ Common variants in *CYP1A2* gene reflect its inducibility. *CYP1A2* genetic variations, without the presence of induction (e.g., smoking, concomitant CYP1A2 inducers), have not been demonstrated to clinically alter the activity of CYP1A2 [37]. ^‡^ *CYP3A4* gene shows some genetic variations and most variants have not been demonstrated to clinically alter the activity of CYP3A4. Many of the variants are extremely rare, making it difficult to derive a phenotype based on genetic results [38].

**Table 4:** Summary of binding affinity (strength represented by color gradient) for various CYP isoforms and the percent of drug elimination by these respective CYP metabolic pathways pre- and post-PGx intervention) ^†^.

Pre-PGx Clinical Interventions				Post-PGx Clinical Interventions			
Substance	CYP1A2	CYP2D6	CYP3A4	Substance	CYP1A2	CYP2D6	CYP3A4
Acetaminophen	15%			Acetaminophen	15%		
Amlodipine			30%	Amlodipine			30%
Apixaban			50%	Apixaban			50%
Duloxetine	70%	30%		Desvenlafaxine	NON-CYP		
Dronedarone			84%	Dronedarone			84%
Hydrocodone		10% ^‡^	40%	Morphine	NON-CYP		
Loperamide			65%	Loperamide			65%
Melatonin	60%			Melatonin	60%		
Metoprolol		75%		Carvedilol		60%	
Ondansetron	20%		35%	Ondansetron	20%		35%

Abbreviations: CYP: Cytochrome P450, PGx: Pharmacogenomics. ^†^ Only CYP-metabolized oral drugs are displayed. ^‡^ Prodrug. Legend: 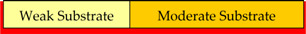 MedWise^®^ depicts the various degrees of binding affinity of a substrate for a specific enzyme using different colors, such as: light yellow (weak affinity) and dark yellow (moderate affinity). The percentages listed correspond to the use of the metabolic pathway for the substrate.

## Data Availability

The data presented in this study are available in the article.

## Supplementary Materials

The following are available online at https://www.mdpi.com/article/10.3390/medicina57090955/s1, Table S1: Adapted CPIC Recommendations to Guide Hydrocodone Therapy Considering CYP2D6 Phenotype, Table S2: Adapted DPWG Recommendations to Guide Metoprolol Therapy Considering CYP2D6 Phenotype, Table S3: Adapted DPWG Recommendations to Guide Duloxetine Therapy Considering CYP2D6 Phenotype. References [58,59] are cited in the supplementary materials.
