# Supplementary material for: Pharmacist-Led Medication Evaluation Considering Pharmacogenomics and Drug-Induced Phenoconversion in the Treatment of Multiple Comorbidities: A Case Report
Source: Medicina (Kaunas). 2021 Sep 10;57(9):955. doi: 10.3390/medicina57090955 (PMC8466444; doi:10.3390/medicina57090955)
Supplement: Supplementary file 1 [file medicina-57-00955-s001.zip › medicina-1358135-supplementary.pdf]

Supplementary Materials:

Table SI. Adapted CPIC Recommendations to Guide Hydrocodone Therapy Considering CYP2D6 Phenotype[11]

CYP2D6 Phenotype.	Implications and Clinical Interpretation	Recommendations
**Ultra-Rapid Metabolizer**	Minimal evidence for pharmacokinetic or clinical effect.	No recommendation for hydrocodone therapy because of minimal evidence regarding adverse events or analgesia.
**Normal Metabolizer**	Normal hydromorphone formation.	Use hydrocodone label recommended age- or weight-specific dosing.
**Intermediate Metabolizer**	Minimal evidence for pharmacokinetic or clinical effect.	Use hydrocodone label recommended age- or weight-specific dosing. If no response and opioid use is warranted, consider non-codeine or non-tramadol opioid.
**Poor Metabolizer**	Decreased metabolism of hydrocodone to active metabolite hydromorphone, but there is insufficient evidence to determine if these effects on pharmacokinetics translate into decreased analgesia or side effects.	Use hydrocodone label recommended age- or weight-specific dosing. If no response and opioid use is warranted, consider non-codeine or non-tramadol opioid.

Abbreviations: CPIC: Clinical Pharmacogenetics Implementation Consortium; CYP: Cytochrome P450

Table SII. Adapted DPWG Recommendations to Guide Metoprolol Therapy Considering CYP2D6 Phenotype[58]

CYP2D6 Phenotype.	Implications and Clinical Interpretation	Recommendations
**Ultra-Rapid Metabolizer**	The gene variation increases the conversion of metoprolol to inactive metabolites. This can increase the dose requirement. However, with a target dose of 200 mg/day, there was no effect on the blood pressure and hardly any effect on the reduction of the heart rate.	Use the maximum dose for the relevant indication as a target dose. If the effectiveness is still insufficient:<br>Increase the dose based on effectiveness and side effects to 2.5 times the standard dose or select an alternative.<br>Possible alternatives include:<br>• Heart failure: bisoprolol or carvedilol.
**Intermediate Metabolizer**	The gene variation reduces the conversion of metoprolol to inactive metabolites. However, the clinical consequences are limited mainly to the occurrence of asymptomatic bradycardia.	If a gradual reduction in heart rate is desired, or in the event of symptomatic bradycardia:<br>Increase the dose in smaller steps and/or prescribe no more than 50% of the standard dose.
**Poor Metabolizer**		If a gradual reduction in heart rate is desired, or in the event of symptomatic bradycardia:

		Increase the dose in smaller steps and/or prescribe no more than 25% of the standard dose.

Abbreviations: DPWG: Dutch Pharmacogenetics Working Group; CYP: Cytochrome P450

Table SIII. Adapted DPWG Recommendations to Guide Duloxetine Therapy Considering CYP2D6 Phenotype[59]

CYP2D6 Phenotype.	Implications and Clinical Interpretation	Recommendations
**Ultra-Rapid, Intermediate, or Poor Metabolizer**	Although plasma concentrations of duloxetine may be affected, a typical response is expected.	There are currently no dosing recommendations for duloxetine based on CYP2D6 genotype.

Abbreviations: DPWG: Dutch Pharmacogenetics Working Group; CYP: Cytochrome P450
